# Factors Influencing Spiritual Care for African Americans in Hospice in the United States: An Exploratory Study of the Perspectives of Their Caregivers, Clergy and Hospice Chaplains

**DOI:** 10.1007/s10943-025-02416-1

**Published:** 2025-08-12

**Authors:** Denise D. Quigley, Sara G. McCleskey, Jason Lesandrini, Natalie McNeal, Nabeel Qureshi

**Affiliations:** 1 RAND Corporation, 1776 Main Street, Santa Monica, CA 90401 USA; 2 Wellstar Health System, Marietta, GA USA; 3 Hospice of Northeast Georgia Medical Center, Gainesville, GA USA; 4 RAND School of Public Policy, Santa Monica, CA USA

**Keywords:** Healthcare delivery, Spiritual care, Hospice, Minority population, Chaplains

## Abstract

Caregivers of African American hospice patients report unsatisfactory spiritual support in hospice. Hospice professionals, chaplains, clergy, and policymakers need to understand factors influencing spiritual care of African American hospice patients to advance equitable end-of-life and hospice care. We partnered with large community hospice in Georgia, USA, and interviewed caregivers of African American hospice decedents, their clergy, and chaplains. We heard caregivers of African American hospice patients desired chaplains to pray, visit frequently and be present. Caregivers’ lack of knowledge about hospice, general medical mistrust, and clergy’s minimal understanding of the breadth of spiritual care were barriers to adequate spiritual care.

## Introduction

Spiritual care is the *recognition of* patient and caregiver spiritual and religious needs and *attention to* those needs, as defined by Daaleman (Daaleman & VandeCreek, 2000). Spiritual care promotes positive religious coping, improves symptoms of patient distress (e.g., anxiety, difficulty breathing), and can improve patient and caregiver psychosocial outcomes (e.g., unresolved feelings, distress) (McClement et al., 2007; Pargament, 1997; Pargament et al., 1998; Rippentrop et al., 2005) which are tied to caregiver strain (Andrews, 2001; Bedard et al., 2001; Pearlin et al., 1990; Whitlatch et al., 1991; Zarit et al., 1980) or grief (Anderson et al., 2008; Goldsmith et al., 2008; Gries et al., 2010; Kiely et al., 2008; Maciejewski et al., 2007). Spiritual care is an integral part of hospice care (Flannelly et al., 2003; VandeCreek & Lyon, 1994/1995).

According to the National Hospice and Palliative Care Organization, a smaller proportion of African Americans are hospice decedents (i.e., 10.7% of hospice decedents are African American) than the national proportion of Black individuals in the USA (i.e., 13% of US population is African American) (Estrada et al., 2021; National Hospice and Palliative Care Organization, 2024; United States Census Bureau, 2022), indicating lower hospice utilization by African Americans. Also, caregivers of African American hospice patients (compared to those of White hospice patients) report receiving less information on patient symptoms, worse care coordination in hospice (Rhodes et al., 2007; Welch et al., 2005), and worse emotional, spiritual, and religious support (Astrow et al., 2018; Parast et al., 2021; Price et al., 2017). A recent study found that clergy and chaplains have distinct, complementary roles in providing spiritual care to African American hospice patients and families (LeBaron et al., 2016; Quigley et al., 2024), stating that both clergy and chaplains are needed to provide desired spiritual care for African American hospice patients and their caregivers (Balboni et al., 2013; Quigley et al., 2024), Yet, *how* spiritual care for African American patients is worse and what factors influence this spiritual care are not well understood.

Spiritual care in hospice could be influenced by patient or caregivers’ lack of trust in healthcare providers (Crawley et al., 2000; Institute of Medicine (US) and National Research Council (US) National Cancer Policy Board, 2001; Sewell, 2015), African American pastors’ unfamiliarity with hospice (Johnson et al., 2005, 2019; Reese et al., 1999) or system factors such as perceived/experienced discrimination, lack of racial concordance or limited culturally competent spiritual care by hospice chaplains. Also, since African Americans are the most religious racial and ethnic group in the USA (Masci, 2018), African American hospice patients and their caregivers may have different patterns of where they seek spiritual care and what they desire than other hospice patients and caregivers.

Developing a better understanding of what constitutes spiritual care and emotional support in hospice for African American patients and the circumstances that influence receiving the right amount of spiritual care is necessary to both eliminate the current racial disparities in spiritual care in hospice (National Institute of Nursing Research, 2016) and inform interventions to improve spiritual care (Parast et al., 2021). We examine the perspectives of African American caregivers of hospice decedents, their clergy and chaplains to delineate what behaviors and interactions are described as the right amount of spiritual care and emotional support for African American hospice patients and their caregivers. We also describe the factors related to providing spiritual care for African American hospice patients and their caregivers.

## Methods

*Setting.* This pilot study was conducted in a large, urban/suburban community hospice within a health system in Georgia in the USA that provides hospice care at home, within a general inpatient unit at numerous hospitals, and in two free-standing hospital inpatient units (each with 18 beds). The hospice employs two full time physicians (board-certified in palliative care), 80 registered nurses, four licensed practical nurses, nine social workers, and five chaplains for roughly 1,700 patients annually. Since 2014, the hospice employs a quality monitoring system based on the Consumer Assessment of Healthcare Providers and Systems (CAHPS) Hospice survey. Detailed methods are described elsewhere (Quigley et al., 2024).

*Sample.* In 2023, the hospice identified bereaved caregivers of African American hospice decedents who died from October to December 2022 (*n* = 58). During routine 90-day bereavement outreach calls, the hospice bereavement coordinator provided information about the study and asked whether caregiver would allow the hospice to share their contact information for study recruitment. Forty percent (23/58) of caregivers agreed.

Chaplaincy staff at the hospice (*n* = 5) were provided an advance letter via email about the research study and asked whether the hospice administrator could provide their contact information to the research team for further follow-up. Community clergy who supported the decedents were identified during caregiver interviews. The interviewer asked whether the caregiver was willing to share clergy contact information for recruitment into the study. After obtaining clergy contact information, the study team contacted clergy about participation in an interview.

*Data Collection.* We conducted 20 h-long semi-structured phone interviews with chaplains employed at the hospice (*n* = 5); bereaved caregivers of African American decedents who died receiving hospice care (*n* = 12); and their clergy who ministered directly to the identified bereaved caregivers or decedents during hospice care (*n* = 3). Interviews were conducted by the lead author with a second member of the research team taking detailed notes.

Bereaved caregivers were asked about their general perceptions of hospice; practical help; emotional support; religious or spiritual support; and any issues experienced with hospice. Caregivers were asked the three CAHPS Hospice survey questions verbatim related to emotional support and to religious and spiritual support. We also asked about the amount of religious and spiritual support the decedent received on hospice. For these questions, we probed on whether it was too little, the right amount, or too much to replicate the CAHPS Hospice 3-point response scale. Adequate support/care was defined by the caregiver reporting “the right amount” response to these questions; inadequate was defined by “too little”) (see Table 2 for question wording).

Chaplains were asked about their experiences providing spiritual care at the hospice, providing spiritual care to diverse communities, and interactions with community clergy. Clergy were asked about experiences working in hospice and their interactions with the bereaved caregiver and decedent during their time in hospice. Interview protocols are available upon request. Interviews were recorded and transcribed verbatim.

*Analytic Approach.* We entered transcripts into Dedoose (SocioCultural Research Consultants, 2018; SocioCultural Research Consultants, 2019), a web application for analyzing qualitative data. We established initial codes using the deductive process of creating codes that mapped to key research questions (Bernard & Ryan, 2010) and to literature on emotional, spiritual, and religious support in hospice and care disparities at the end of life (Daaleman et al., 2008a, 2008b; Edwards et al., 2010; Hvidt et al., 2020; LeBaron et al., 2016). We further developed codes using inductive procedures generating codes from insights from transcripts (Bradley et al., 2007; Thomas, 2003). We used this same coding process for each data source (i.e., caregiver interviews, chaplain interviews, clergy interviews, and decedent medical charts).

Three researchers conducted coding to identify topics, coding early transcripts independently and refining the codebook (Bernard & Ryan, 2010). We employed interrater reliability exercises among the coding team coding the same transcript to refine codes and descriptions. We obtained a pooled kappa coefficient of 0.89 for chaplains, 0.86 for caregivers; and 0.91 for clergy, all indicating “very good” agreement. We employed ongoing training for coders on emerging sub-codes using the Dedoose training module. Remaining transcripts were divided among the coders and coded independently. We used regular team meetings to reach consensus on topics, identify discrepancies, refine concepts, codebook changes, define codes and dialogue about themes (Miller & Crabtree, 1999).

We conducted thematic analysis (Braun & Clarke, 2012) to identify themes and key factors to providing spiritual care in hospice. We identified caregivers who reported adequate/inadequate spiritual care and adequate/inadequate emotional support notating how and what was described as adequate and inadequate spiritual care and emotional support.

Study protocols were approved by RAND’s Human Subjects Protection Committee (IRB Assurance No.: FWA00003425;IRB_No.: IRB00000051; Project ID:2023-N0018). All participants provided verbal informed consent prior to interviews.

## Results

*Chaplain and clergy characteristics*. Table 1 lists chaplain (*n* = 5) and clergy (*n* = 3) characteristics. We interviewed all five chaplains on staff at the participating hospice and all three clergy who had visited the 12 African American hospice patients. Clergy and chaplains were predominantly male. All chaplains identified as White, and all clergy identified as African American. Most Clergy and chaplains identified as Baptist. Clergy and chaplains both had a mean age near 60. Chaplains on average had 15.5 years of hospice experience and seven years working at the participating hospice. Most chaplains also had experience working in another hospice. Clergy had on average 22 years of experience in ministry as pastors.

**Table 1:** Participant demographic characteristics of hospice chaplains (*N* = 5) and community clergy (*N* = 3)

Characteristic	Hospice chaplain (*n* = 5)	Community clergy (*n* = 3)
	*Mean (Range)*	*Mean (Range)*
Age	58.8 years (42–70)	60.3 years (42–75)
Years in hospice care	15.5 years (7.5–26)	NA
Years at named hospice	7.1 years (3–11)	NA
Years as a pastor	NA	22.3 years (17–30)
	*% (N)*	*% (N)*
Male	80% (4)	100% (3)
Race		
Percent White	100% (5)	0% (0)
Percent African American	0% (0)	100% (3)
Religious Background		
Non-denominational Christian	20% (1)	33% (1)
Baptist	60% (3)	67% (2)
Methodist	20% (1)	0% (0)
Worked at another hospice	80% (4)	NA

*Caregiver-reported support in hospice*. Table 2 provides caregiver-reported emotional support and religious and spiritual support provided to the caregiver and decedent (*n* = 12). Most caregivers reported the emotional support they received in hospice and after was adequate (i.e., right amount; *n* = 8). Most caregivers reported that the religious and spiritual support that they and the patient received was adequate (*n* = 7).

**Table 2:** Caregiver-reported emotional support of caregiver in hospice and after death and religious and spiritual support of caregiver and of the patient in hospice (*N* = 12)

*n* = 12	Emotional support of caregiver in hospice	Emotional support of caregiver after death	Religious and spiritual support of caregiver in hospice	Religious and spiritual support of patient in hospice
Question Wording	While your family member was in hospice care, how much emotional support did you get from the hospice team?	In the weeks after your family member died, how much emotional support did you get from the hospice team?	While your family member was in hospice care, how much support for your religious and spiritual beliefs did you get from the hospice team?	While your family member was in hospice care, how much support did they receive for their religious and spiritual beliefs from the hospice team?
CAHPS hospice item	Yes	Yes	Yes	No
Too little	3	4	5	5
Right amount	9	8	7	7
Too much	0	0	0	0
Adequate emotional support (i.e., Right Amount for Emotional Support items)	9	8	*Not Applicable*	*Not Applicable*
Adequate spiritual care (i.e., Right Amount for Religious and Spiritual Support questions)	*Not Applicable*	*Not Applicable*	7	7

*Decedent and caregiver characteristics*. Table 3 provides decedent and caregiver characteristics overall (*n* = 12) and by whether the caregiver-reported adequate (*n* = 7) or inadequate (*n* = 5) spiritual care. On average, decedents were 74.8 years old. Comparing those reporting adequate versus inadequate spiritual care, those who reported adequate spiritual care were older on average (78.0 vs. 69.7, respectively). Decedents spent almost 2 weeks on hospice on average (13.8 days); this length of stay on hospice was similar for those reporting adequate and inadequate spiritual care. Most decedents were non-denominational Christian or Baptist; the majority of those reporting adequate spiritual care were Baptist, whereas the majority reporting inadequate spiritual care were non-denominational Christian (67% and 83%). Two-thirds expired in the inpatient hospice unit; the remainder died at home. Decedents were mostly parents of their caregivers, followed by spouses/fiancés. Almost all wanted chaplain care (92%), three-fourths were offered it, and only two-thirds received it. Caregivers who reported adequate spiritual care received chaplain care more often than those who reported inadequate spiritual care (71% and 60%). All who reported inadequate care wanted chaplain care, and three-fifths were offered it and got it.

**Table 3:** Participant demographic characteristics of African American decedents and their caregivers, overall (*N* = 12) and by whether caregiver-reported adequate (*N* = 7) or inadequate (*N* = 5) spiritual care

Characteristic (and Source)	Overall African American decedents (*n* = 12)	African American decedents whose caregivers reported *Adequate* spiritual care in hospice (*n* = 7)	African American decedents whose caregivers reported *Inadequate* spiritual care in hospice (*n* = 5)
	Mean (SD)	Mean (SD)	Mean (SD)
Decedent age in years	74.8 (14.3)	78.0 (5.5)	69.7 (8.8)
Time in hospice in days (from EHR)	12.9 (7.3)	13.2 (7.4)	11.8 (0.1)
Time in hospice in days (from interview)	13.8 (10.4)	13.7 (7.9)	13.8 (6.6)
	% (n)	% (n)	% (n)
Decedent religion (from interview)			
Non-denominational Christian	42% (5)	14% (1)	80% (4)
Baptist	33% (4)	57% (4)	0% (0)
Methodist	8% (1)	0% (0)	20% (1)
Jehovah’s Witness	17% (2)	29% (2)	0% (0)
Decedent discharge disposition (from EHR)			
Expired in inpatient hospice unit	67% (8)	71% (5)	60% (3)
Expired at home	33% (4)	29% (2)	40% (2)
Relationship to caregiver (from EHR)			
Parent	50% (6)	57% (4)	40% (2)
Spouse/Fiancé	33% (4)	29% (2)	40% (2)
Sibling	8% (1)	0% (0)	20% (1)
Friend	8% (1)	14% (1)	0% (0)
Chaplain care (from interview)			
Offered chaplain care	75% (9)	86% (6)	60% (3)
Wanted chaplain care	92% (11)	86% (6)	100% (5)
Got chaplain care	67% (8)	71% (5)	60% (3)

Adequate indicates it was the “right amount”; inadequate indicates it was “too little.”

*Spiritual care themes*. Several themes emerged regarding spiritual care provided to African American hospice patients and caregivers. Table 4 provides the main themes and subthemes regarding the amount of spiritual care and emotional support in hospice (Themes #1 and #2) and the factors influencing spiritual care (Themes #3 and #4). Appendix 1 provides illustrative quotes for the themes #1 and #2, and Appendix 2 provides illustrative quotes for themes #3 and #4.

**Table 4:** Themes, subthemes for spiritual care, emotional support and factors influencing spiritual care

Theme	Subtheme
#1: Amount of spiritual care	The right amount of spiritual care included chaplains visiting regularly/frequently, being present, helpful, talking, and praying with the decedents and caregivers
	African American decedents and caregivers identified the same factors (i.e., visiting regularly, being present, helpful, talking, and praying) to determine whether they received the right amount of spiritual care
	Some African American families reported not needing spiritual support from the hospice because it was provided by the family
	Too little spiritual care included not getting spiritual care from the chaplain
	Inadequate spiritual care also included not getting enough time with the chaplain or that the chaplain’s approach did not match the decedent’s or caregiver’s preferences
#2: Amount of emotional support	The right amount of emotional support for African American decedents included nurses and social workers being attentive, available, talking and showing dignity to the decedent
	The right amount of emotional support for caregivers included nurses following-up with practical steps on how to access offered hospice services (e.g., counseling) and regular contact after decedent passed including bereavement services
	Too little emotional support for caregivers included a lack of wanted contact, information and detailed follow-up
#3: Medical mistrust	Chaplains echoed that medical mistrust exists among African American hospice patients and were aware of racism and historical harms to African American patients broadly and also for hospice patients specifically
	Lack of trust in the medical system generally leads to a lack of trust in hospice staff and facilities
#3: Discrimination	Most caregivers perceived that racial and discrimination issues were most often experienced in the hospital setting, and while they were not repeated in their recent hospice experience, they still resonated with the fears and mistrust
	Even if discrimination is not overt, discrimination can lead to medical mistrust and substandard care
	Chaplains reported instances in which patients discussed feelings of discrimination in other care settings that impact spiritual care for African American patients
#3: Sharing race with care team	Most caregivers perceived that racial and discrimination issues were most often experienced in the hospital setting and were not repeated in their recent hospice experience
	For caregivers, sharing race or ethnicity with their providers leads to better treatment
#4: Lack of knowledge about hospice	Chaplains pointed to several typical pre-conceived notions about hospice that they heard from African Americans. These included that hospice is somewhere where you die, that it is where you are sent when the family or caregivers have given up on the patient, and /or that spiritual care at the end of life is only religious support
	Chaplains reported that African Americans caregivers often expressed hesitancy to spiritual care related to the caregiver’s lack of knowledge about hospice and not understanding the value hospice or chaplains can provide
	African American clergy also had minimal understanding about the breadth of spiritual care in hospice at end of life. Education about hospice was raised as important not just for caregivers and patients, but also for African American clergy to ensure that everyone involved in hospice understands the role of hospice in end-of-life care and can make informed decisions about hospice services, settings, etcetera
	Education for African American patients and families about hospice services and care at end of life was discussed as not needing to be formal and could be accomplished through word of mouth

Illustrative quotes for each theme and subtheme are provided in the Appendix 1 Table or Appendix 2 Table

*Theme#1: The right amount of spiritual care included chaplains being present, helpful, talking, praying, and visiting regularly/frequently. Caregivers who reported too little spiritual care wanted a chaplain or desired more chaplain visits and prayer, someone who would walk alongside them.*

Caregivers described the right amount of spiritual care they received as the chaplain visiting regularly, being present, helpful, talking and praying. Similarly, the same aspects of spiritual support by chaplains—frequent/regular visits, presence, and prayer—were described as the right amount for the African American decedents. The only exceptions were the few caregivers who indicated that their family was the spiritual support that the decedent needed so they did not need spiritual support from the hospice.

Caregivers that indicated they received too little spiritual care described their care and what they wanted. Several caregivers indicated what the hospice provided was too little because they wanted a chaplain and did not get one. Other caregivers expressed that they wanted the chaplain more often, someone to walk alongside them. Other caregivers described the chaplain’s approach as lacking. Caregiver also described what they meant by wanting more spiritual support. This included providing education on the services they could provide, or written materials the caregivers or decedents could use as a reference.

*Theme#2: The right amount of emotional support for African American decedents included nurses and social workers being attentive, available, talking and showing dignity. For the caregivers themselves, the right amount of emotional support included nurses following-up with practical steps on how to access offered hospice services (e.g., counseling) and regular contact after decedent passed.*

Caregivers described nurses and social workers providing the right amount of emotional support for their loved ones in terms of them being attentive, available, talking and showing dignity to the decedent. Caregivers described the support they received from the hospice as being helpful and practical. Caregivers described the emotional support offered in bereavement.

Caregivers that indicated they received too little emotional support described what they wanted including more contact, information, and detailed follow-up. Caregiver pointed out their need for information to feel emotionally supported. Caregivers explained their need for more detailed follow-up pertaining to hospice services.

*Theme#3: Chaplains echoed that medical mistrust exists among African American hospice patients and is a barrier to spiritual care unless patient/caregivers understand the non-medical emotional/spiritual support role of chaplains. Caregivers of African American hospice patients discussed how race can be an important element in hospice and how sharing the same race with the care team affects trust.*

Chaplains noted that they have to be aware of racism and historical harms to African American patients broadly and also for hospice patients specifically. They pointed out that racism and historical harm extends beyond the healthcare system. Chaplains reported experiencing and recognizing that African American hospice patients have reason to mistrust the medical system due to historical discrimination. While discrimination is no longer overt, it can lead to substandard care.

Chaplains discussed how a lack of trust in the medical system generally leads to a lack of trust in hospice staff and facilities. Often, these concerns, chaplains explained, are substantiated within their communities. Caregivers noted also that race does matter in how providers treat hospice patients. In their view, sharing race or ethnicity with their providers leads to better treatment. Most caregivers perceived that racial and discrimination issues were most often experienced in the hospital setting, and while they were not repeated in their recent hospice experience, they still resonated with the fears and mistrust.

Chaplains noted that African Americans often believe that hospice is the same as giving up on their loved ones. Chaplains also reported instances in which patients discussed feelings of discrimination. Again, these sentiments were most often about other settings of care in the healthcare system, and did not include spiritual care providers. However, such context of healthcare is important as it is lived experience of African Americans.

*Theme#4: Chaplains reported that African Americans caregivers often expressed hesitancy to spiritual care related to the caregiver’s lack of knowledge about hospice and not understanding the value hospice or chaplains can provide. African American clergy also had minimal understanding about the breadth of spiritual care in hospice at end of life.*

According to chaplains, African American patients, their families and clergy have a limited understanding and knowledge of hospice services and care, particularly in understanding what it is, what it is not, and how it can be helpful at the end of life. Chaplains indicated that main, typical pre-conceived notions of hospice that they heard from African Americans included that hospice is somewhere where you die, that it is where you are sent when the family or caregivers have given up on the patient, and /or that spiritual care at the end of life is only religious support. Chaplains noted that their role in hospice care is sometimes understood incorrectly.

Education about hospice was raised as important not just for caregivers and patients, but also for African American clergy to ensure that everyone involved in hospice understands the role of hospice in end-of-life care and can make informed decisions about hospice services, settings, etc.. One chaplain noted that this type of education could “*help get rid of the fear and distrust*” that many African American patients, caregivers, and clergy may feel about hospice, that stems either from distrust of the medical system overall, or from fear that hospice is “*trying to kill you*” (Chaplain#1). This same type of education would also help nurses who also work on integrating spiritual care into end-of-life care. Another chaplain noted that “*the more training, the better for nurses, for patients, for families on what is spiritual care, what is chaplain role in hospice*” (Chaplain#5).

Education on hospice services and care at end of life was discussed as not needing to be formal and could be accomplished through word of mouth. Clergy and African American patients or families with good experiences with hospice could potentially be a trusted source of information about hospice and be in the forefront of dispelling misunderstandings about hospice and building trust in the process leading up to and including hospice care.

## Discussion

We found that most caregivers of African American hospice patients reported receiving the right amount of spiritual care and emotional support for their loved one and themselves. They also described spiritual care as distinct from emotional support. Caregivers reported their desired spiritual care to include chaplains being present, praying, and visiting regularly/frequently (i.e., every day or every other day). Also, caregivers described the emotional support provided to their loved one as nurses/social workers being attentive, available, talking and showing dignity. Caregivers described the emotional support they received primarily as follow-through on requests and regular contact in bereavement. These distinctive behaviors by different hospice care team members included in spiritual care and emotional support highlight the need to measure/track emotional support and spiritual care provided by hospices separately to better understand the disparities in care for African Americans. Further investigation on the frequency of these behaviors for African American patients and caregivers is also warranted.

Lack of knowledge about hospice, primarily concerning the role of chaplains and the breadth of spiritual care and support available in hospice among African American caregivers and clergy were reported as barriers to providing adequate spiritual care. We heard from caregivers with and without previous hospice experience that they were reticent to participate in spiritual care without more information about the chaplain’s role and how it could help. Those that had previous experience with hospice had not found the chaplain helpful or present enough; Those without hospice experience were “leery” of hospice without knowing more about what additional support chaplains could provide. Lack of knowledge about hospice was commonly expressed by caregivers and also identified by chaplains as a barrier to providing spiritual care. These findings are not novel as research indicates that African Americans have limited knowledge or familiarity with end-of-life discussions, advance care planning, and hospice (Johnson et al., 2019), pointing to both incongruent information and inconsistent sources of health care information (Daaleman et al., 2008a, 2008b) as a main reason. Efforts have focused on diversifying hospice staff, building community partnerships to create culturally relevant materials (Elk et al., 2020), and providing an advance directive coach (Skolarus et al., 2021); yet, more is needed (Robinson, 2022).

In our study, African American clergy also acknowledged having minimal understanding about the breadth of spiritual care and support provided in hospice, which aligns with what is known concerning African American pastors being unfamiliar with hospice (Johnson et al., 2005, 2019; Reese et al., 1999). As a result, there is a need to identify processes for how to engage more with clergy. Attention needs to be paid by hospice organizations, leaders and care teams on how to better integrate the spiritual care and work of community clergy for the betterment of all hospice patients and families, but especially for African Americans, who are more likely to have clergy outside of hospice since they are the most religious racial and ethnic group in the US (Masci, 2018).

General medical mistrust by African Americans was recognized as important issue to address by hospice care team members and resonated with chaplains caring for African Americans in hospice; this finding of general medical mistrust by African Americans is supported by other evidence concerning patient or caregiver lack of trust in providers in healthcare more generally, that is, not specific to hospice care (Crawley et al., 2000; Institute of Medicine (US) and National Research Council (US) National Cancer Policy Board, 2001; Sewell, 2015). However, trust is an important component of communication, connectedness, empathy, and showing respect, all of which are necessary components for provider-patient communication. Efforts to bolster trust and connectedness that support chaplain and hospice team communication with caregivers and patients are important to having robust spiritual care programs in hospice.

Additionally, we did not hear consistent themes from caregivers, clergy or chaplains about other system factors such as perceived/experienced discrimination, lack or need for racial concordance with hospice professionals, or a lack of culturally competent spiritual care from hospice chaplains. Further investigation of these system factors within hospice is warranted.

*Limitations*. Our study has limitations. We explored and examined one hospice organization’s experience with spiritual care provided by chaplains and clergy to African American hospice patients and their caregivers and our sample sizes were small, limiting generalizability of our findings. However, our study design of gaining the perspectives of each African American hospice patient’s caregiver, their clergy, and hospice chaplain allowed us a comprehensive, in-depth picture of their hospice experience and the roles of chaplain and clergy in emotional, spiritual and religious support. Our findings on the distinctive behaviors and needs included in spiritual care and emotional support raise important issues for spiritual care delivery in hospice for all patients and highlight the need for further investigation. As an exploratory study, we could not include a larger sample of caregivers or clergy or interview other interdisciplinary hospice team members (i.e., nurses), nor include a comparison group of White hospice decedents. Thus, a broader, multi-site evaluation of hospices caring for African American hospice patients and caregivers that includes a comparison group and interviews with the full interdisciplinary hospice team may identify additional insights to better understand reasons for disparities and avenues for improving the quality of hospice spiritual care.

## Conclusion

Lack of knowledge about chaplains and the breadth of spiritual care and support available among African American caregivers and clergy continue to be barriers to spiritual care in hospice. Yet, caregivers of African American hospice patients expressed the desire for chaplains to pray, visit frequently and be present. This desired spiritual care was distinct from the emotional support needed from the broader interdisciplinary team, highlighting the distinct and important behaviors of chaplain and clergy in providing adequate spiritual care in hospice for African Americans. Hospice professionals, chaplains, community clergy, and policy makers need to understand the array of factors influencing spiritual care of African Americans in hospice care to advance equitable end-of-life and hospice care for all hospice patients.

## Data Availability

The data from the study are unavailable due to confidentiality.

## Appendix 1

See Table 5.

**Table 5:** Themes, subthemes and illustrative quotations for spiritual care and emotional support (Themes #1 and #2)

Theme	Subtheme	Illustrative quotes
Amount of spiritual care	The right amount of spiritual care included chaplains visiting regularly/frequently, being present, helpful, talking, and praying with the decedents and caregivers	Yes, it was the right amount. We had the chaplain. The chaplain offered to pray with us, sat with us. We were on the same page spiritually.–Caregiver#0202
	African American decedents and caregivers identified the same factors (i.e., visiting regularly, being present, helpful, talking, and praying) to determine whether they received the right amount of spiritual care	[DECEDENT] did talk to the chaplain. She was open to speaking with the chaplain and the chaplain prayed with her; that helped her quite a bit, to exercise her faith.–Caregiver#0108 Chaplain came by twice before my wife passed. Chaplain talked to both of us and gave me the Daily Bread, the book of daily scripture readings. The chaplain was there with us. Chaplain mostly talked to us and did pray with us.–Caregiver#0111
	Some African American families reported not needing spiritual support from the hospice because it was provided by the family	We [DECEDENT]’s family were there for [DECEDENT] for emotional, spiritual support. So, it was the right amount for him as we did not need that from hospice.–Caregiver#0104
	Too little spiritual care included not getting spiritual care from the chaplain	Too little spiritual support. They didn’t offer a chaplain. They offered counseling. I would have liked to talk with a chaplain.–Caregiver#0106 I don’t have anything to compare it to, but I would have to say too little [religious spiritual support] because of the 2 or 3 requests for support that were not met. I asked for a chaplain to come, respite so I could grocery shop, and a grief group. I don’t think I asked for a lot, but hospice did not provide any of them. It was too little.–Caregiver#0110
	Inadequate spiritual care also included not getting enough time with the chaplain or that the chaplain’s approach did not match the decedent’s or caregiver’s preferences	Hospice spiritual support was too little. It would have been better had [DECEDENT] also had a chaplain visiting regularly, to walk alongside them during this part of their life.–Caregiver#0101 It was too little [religious spiritual support], it was just the chaplain coming once to pray. The chaplain came, but not enough.–Caregiver#0113 The [religious spiritual support] by the hospice was too little. There is something about presence, being there, coming to be with my dad in his room…..that would have helped. My dad would have wanted prayer, someone there with him. The chaplain only stopped by, introduced himself. It was a little cookie cutter for me. Kind of vanilla. The chaplain said here is my number, let me know if you need prayer and left. It was too little.–Caregiver#0121 The chaplain coming sooner would have been a help to [DECEDENT]. Even if the chaplain had material, something to read to him that would have been better. I think the chaplain only gave me a business card. Definitely too little.–Caregiver#0122
Amount of emotional support	The right amount of emotional support for African American decedents included nurses and social workers being attentive, available, talking and showing dignity to the decedent	The emotional support was the right amount. The attending nurses were comforting and would ask if I needed anything. They were attentive to her. They would also talk to her and recognizing she was still there and a human being. They would talk and ask if I needed anything, and said I could come any time of the day. They also said I could ask for anything.–Caregiver#0108
	The right amount of emotional support for caregivers included nurses following-up with practical steps on how to access offered hospice services (e.g., counseling) and regular contact after decedent passed including bereavement services	We got the right amount of support. The hospice made it clear they were just a phone call away. One time I called them to get help. They walked me through everything, stayed on the phone with me. They told me the nurse could come if we needed it, or they could reach out to our RN as well.–Caregiver#0202 We were contacted on several occasions from hospice–immediately after he passed they called and 3 weeks to a month after they called. They also sent cards. They offered meetings, grief counseling providing the numbers and steps to access them. It was not pushy, it was the right amount of support.–Caregiver#0114
	Too little emotional support for caregivers included a lack of wanted contact, information and detailed follow-up	In terms of emotional support, I wish they would have called me. They should have called me. I want to be fair. They did call. Not often. I don’t know how much I would want at that time. They did what they could. But had they called more often that would have been better.–Caregiver#0120 I think it was too little emotional support. I wish they could have been upfront with me more about my husband’s condition. More information would have helped me know what was coming and been a support. No one talked to me about it.–Caregiver#0113 I think the emotional support provided was too little. The social worker or nurses didn’t really talk to me about how to get the grief counseling. They only offered me counseling, but that was about it, no follow-up. So, it never happened.–Caregiver#0106

## Appendix 2

See Table 6.

**Table 6:** Themes, subthemes, and illustrative quotations for factors influencing spiritual care (Theme #3 and Theme #4)

Theme	Subtheme	Illustrative quotes
Medical mistrust	Chaplains echoed that medical mistrust exists among African American hospice patients and were aware of racism and historical harms to African American patients broadly and also for hospice patients specifically	I as a chaplain have to understand racism exists and how the patient or family have been treated by white people, by the healthcare system, the criminal justice system; there is distrust there.–Chaplain#2
	Lack of trust in the medical system generally leads to a lack of trust in hospice staff and facilities	My experiences resonate with the fact that African Americans have a lack of trust in healthcare, that translates into lack of trust in hospice. African Americans also have previous experiences of discrimination for themselves and within their community.–Chaplain#1
Discrimination	Most caregivers perceived that racial and discrimination issues were most often experienced in the hospital setting, and while they were not repeated in their recent hospice experience, they still resonated with the fears and mistrust	Outside of hospice care, mostly when in the hospital, I feel like I can trust the healthcare providers more if we share the same racial background. In hospice, though, that is not what I saw. I think it does not matter in hospice, or didn’t in my experience with [hospice decedent].–Caregiver#0120_inadequate
	Even if discrimination is not overt, discrimination can lead to medical mistrust and substandard care	In African American culture, there is a distrust of the medical field at large because of abuses in the system. Tuskegee is one, but even today, there is substandard health treatment. I can’t blame folks for being leery.–Chaplain#2
	Chaplains reported instances in which patients discussed feelings of discrimination in other care settings that impact spiritual care for African American patients	In one encounter I can remember where an African American patient said they had been discriminated against earlier in their life by the healthcare system, but they told me they didn’t see me as a chaplain as part of that system. They were able to separate me from their earlier experiences of distrust in the healthcare system. They didn’t put me as a chaplain and someone overseeing and offering spiritual care with the medical team who they were discriminated by.–Chaplain#3
Sharing race with care team	Most caregivers perceived that racial and discrimination issues were most often experienced in the hospital setting and were not repeated in their recent hospice experience	Outside of hospice care, mostly when in the hospital, I feel like I can trust the healthcare providers more if we share the same racial background. In hospice, though, that is not what I saw. I think it does not matter in hospice, or didn’t in my experience with [hospice decedent].–Caregiver#0120_inadequate
	For caregivers, sharing race or ethnicity with their providers leads to better treatment	In the hospital, there is a higher level of attentiveness when you share a racial background with the care providers, and they attend better to you.–Caregiver#0120_inadequate
Lack of knowledge about hospice	Chaplains pointed to several typical pre-conceived notions about hospice that they heard from African Americans. These included that hospice is somewhere where you die, that it is where you are sent when the family or caregivers have given up on the patient, and /or that spiritual care at the end of life is only religious support	Many African Americans are angry at the [end of life] situation. If they don’t have someone trustworthy and honest with them, beside them, while they are under care of someone, the anger and not wanting to be on hospice will continue to be a barrier. If they don’t have someone from their community or church to walk along side with them, it is also much harder.–Chaplain#4
		There are a lot of misunderstandings about spiritual care, that we are proselytizing or taking a specific point of view rather than being a comforting presence or a guide… The patients and families just don’t know, or they have a pre-conceived notion.–Chaplain#5
	Chaplains reported that African Americans caregivers often expressed hesitancy to spiritual care related to the caregiver’s lack of knowledge about hospice and not understanding the value hospice or chaplains can provide	We at the hospice aren’t trying to kill you. Hospice aims at comfort. We move alongside the patient and provide them and move them into comfort as they are dying. Hospice provides as much quality of life for the quantity of life that God has left for you and that you can have at the end of life… In the African American culture, there is a distrust of the medical field because of abuses in the system… I can’t blame folks for being leery. People in general don’t know what hospice is about.–Chaplain#1 If [African American patients] don’t have someone trustworthy and honest with them while they are under care of someone, it will continue to be a barrier. If they don’t have someone from their community to walk along side with them, it is harder… For hospice, they think it is where they kill you, and they hate hospice. Education is the answer. And well-trained professionals who address myths and connect bridges… Also using others who are trusted to build that bridge, like a nurse or a pastor who already have a connection. Use these people who have authority and credibility in their life as a foundation to build your relationship on.–Chaplain#1 Sometimes the patients think we are the church police, so we have to make sure they understand we as hospice chaplains are here for emotional support. We aren’t affiliated with the church or a specific religion. We sometimes get a no because they have their own pastor or minister. There are misconceptions about who we are. Often times I utilize the nurse to do a joint visit to show we are more than just someone who is there to scold them. It is a lack of understanding among patients and families of what we as hospice chaplains do.–Chaplain#3
	African American clergy also had minimal understanding about the breadth of spiritual care in hospice at end of life. Education about hospice was raised as important not just for caregivers and patients, but also for African American clergy to ensure that everyone involved in hospice understands the role of hospice in end-of-life care and can make informed decisions about hospice services, settings, etc.	We need more education for clergy in some cases, especially those clergy in the African American church to educate them more about hospice. And some of the lay leaders, like deacons and elders as well. Educating clergy and other church lay staff about what hospice is. What role hospice plays. We at the hospice aren’t trying to kill you. Hospice aims at comfort. We move alongside the patient and provide them and move them into comfort as they are dying.–Chaplain#1
	Education for African American patients and families about hospice services and care at end of life was discussed as not needing to be formal and could be accomplished through word of mouth	It could help if African American patients or families who have had a good experience with hospice would tell others in their community or in their church bodies about the hospice care they received… We need more African American clergy in the community that can… mitigate the 2nd thoughts that people have about hospice.–Chaplain#5
